# Arbovirus‐Associated Guillain–Barré Syndrome: A Systematic Review and Meta‐Analysis of Clinical Characteristics, Subtypes, and Vaccine Associations

**DOI:** 10.1002/iid3.70483

**Published:** 2026-07-06

**Authors:** Eman Taha Osman Ali, Pierre Gashema, Patrick Gad Iradukunda, Emmanuel Edwar Siddig, Claude Mambo Muvunyi

**Affiliations:** ^1^ Faculty of Medical Laboratory Sciences University of Khartoum Khartoum Sudan; ^2^ Department of Research Repolicy Research Centre Kigali Rwanda; ^3^ Department of Drugs Rwanda Food and Drugs Authority Kigali Rwanda; ^4^ Department of Biomedical Service Rwanda Biomedical Centre Kigali Rwanda

**Keywords:** arboviral Infections, co‐infections, Guillain‐Barré syndrome, neurological complications, Zika virus

## Abstract

**Background:**

Arboviral infections are increasingly recognized as triggers of Guillain–Barré syndrome (GBS), yet the clinical spectrum, subtype distribution, and strength of association across different arboviruses remain incompletely characterized. This systematic review and meta‐analysis evaluated the epidemiology, clinical features, and outcomes of arbovirus‐associated GBS.

**Methods:**

PubMed, Scopus, Web of Science, and Google Scholar were searched through early 2025 following PRISMA 2020 guidelines. Observational studies were included in the quantitative meta‐analysis, while case reports and case series were synthesized qualitatively. Random‐effects models were used to estimate pooled prevalence, odds ratios, and clinical outcomes.

**Results:**

One hundred studies were included, comprising 74 case reports/case series, 19 prevalence studies, and seven case–control studies. The pooled prevalence of GBS among individuals with arboviral infection was 1% (95% CI: 0.3%–3.3%), whereas 37% (95% CI: 22%–54%) of patients with GBS had laboratory evidence of recent arboviral infection. Arboviral co‐infections occurred in 16% (95% CI: 8%–31%) of confirmed cases. Case–control studies demonstrated a significant association between Zika virus infection and GBS (OR = 8, 95% CI: 2–34). Qualitative synthesis showed frequent intensive care admission, mechanical ventilation, disability, and mortality. Demyelinating subtypes predominated in Zika virus‐associated GBS, whereas axonal variants were more common following Japanese encephalitis virus infection.

**Conclusions:**

Multiple arboviruses are associated with GBS, with the strongest evidence for Zika virus. Arbovirus‐associated GBS frequently results in severe neurological outcomes, highlighting the need for standardized diagnostics, enhanced surveillance, and prospective multicenter studies to improve understanding of disease mechanisms and optimize patient management.

## 1 Introduction

Guillain–Barré syndrome (GBS) is an acute immune‐mediated disorder of the peripheral nervous system, characterized by rapidly progressive muscle weakness that may lead to respiratory failure and paralysis in severe cases [1]. The condition is commonly triggered by infections, particularly viral pathogens, through immune‐mediated mechanisms such as molecular mimicry, in which host immune responses cross‐react with peripheral nerve components [1, 2]. Additional factors, including host susceptibility, genetic predisposition, and environmental exposures, may further influence disease onset and severity [3].

Arboviral infections have emerged as important infectious triggers of GBS, particularly in regions where these viruses are endemic. Arboviruses, including Zika virus (ZIKV), dengue virus (DENV), chikungunya virus (CHIKV), West Nile virus (WNV), Japanese encephalitis virus (JEV), and tick‐borne encephalitis virus (TBEV), are transmitted by arthropod vectors and have shown increasing geographic expansion driven by globalization, climate change, and vector distribution [4]. Among these, ZIKV gained particular attention during the 2015–2016 epidemic due to its strong association with GBS [5], while DENV and CHIKV have also been increasingly reported as potential triggers [6].

In endemic regions, the co‐circulation of multiple arboviruses presents additional clinical challenges. Co‐infections and prior exposures may influence immune responses, potentially exacerbating disease severity and complicating diagnosis [7, 8, 9]. Furthermore, serological cross‐reactivity and prolonged antibody persistence can hinder accurate identification of recent infections, particularly in areas with widespread vaccination or repeated viral exposure [10, 11].

Despite growing recognition of the link between arboviral infections and GBS, important gaps remain in understanding the clinical spectrum, severity, and subtype distribution associated with different viruses. Variations in host immune responses and viral characteristics may contribute to distinct clinical phenotypes and outcomes; however, these differences have not been systematically synthesized across studies [3, 12].

This systematic review and meta‐analysis aim to comprehensively evaluate the clinical characteristics, Guillain‐Barré Syndrome (GBS) subtypes, and epidemiological associations of arbovirus‐related GBS. Given the rarity and outbreak‐driven nature of arboviral infections, the existing evidence is highly fragmented and predominantly derived from case reports, case series, and heterogeneous observational studies, limiting the ability of individual studies to provide robust or generalizable conclusions. Therefore, a systematic synthesis of the available data is essential to integrate dispersed evidence and provide a more comprehensive understanding of disease patterns. In addition, this study examines the potential impact of co‐infections and vaccine‐related factors on disease presentation and severity. By integrating available evidence, this work seeks to inform clinical practice, improve diagnostic approaches, and highlight key areas for future research. While this review focuses on the most extensively studied arboviruses with global relevance, less‐characterized viruses may also contribute to GBS and warrant further investigation.

## 2 Materials and Methods

### 2.1 Study Design and Search Strategy

This study was conducted as a systematic review and meta‐analysis in accordance with the Preferred Reporting Items for Systematic Reviews and Meta‐Analyses (PRISMA 2020) guidelines [13]. A comprehensive literature search was performed across PubMed, Scopus, Web of Science, and Google Scholar to identify relevant studies published up to early 2025.

The search strategy combined keywords and Medical Subject Headings (MeSH) related to Guillain–Barré syndrome and arboviral infections, including “Guillain‐Barré syndrome,” “arbovirus,” “dengue virus (DENV),” “Zika virus (ZIKV),” “chikungunya virus (CHIKV),” “West Nile virus (WNV),” “Japanese encephalitis virus (JEV),” and “tick‐borne encephalitis virus (TBEV).”

Although predefined methods were followed, the lack of prospective protocol registration may introduce potential bias and limit transparency; therefore, future systematic reviews will ensure prior registration in a recognized database such as PROSPERO.

### 2.2 Eligibility Criteria

Studies were considered eligible if they met the following inclusion criteria: (1) reported confirmed or clinically suspected Guillain–Barré syndrome (GBS) associated with arboviral infections, including dengue virus (DENV), Zika virus (ZIKV), chikungunya virus (CHIKV), West Nile virus (WNV), Japanese encephalitis virus (JEV), or tick‐borne encephalitis virus (TBEV), with or without co‐infections; (2) provided sufficient primary data describing both the arboviral infection and GBS (e.g., diagnostic confirmation, clinical features, or outcomes); and (3) were original research studies, including observational designs (cohort, case–control, cross‐sectional) as well as case series and case reports. All eligible studies published up to early 2025 were considered, without geographic restriction.

Studies were excluded if they met any of the following criteria: (1) review articles, systematic reviews, meta‐analyses, editorials, commentaries, or other non‐primary research; (2) studies lacking sufficient data on either arboviral infection or Guillain–Barré syndrome after full‐text assessment; (3) duplicate publications or overlapping datasets, in which case the most comprehensive study was retained; and (4) studies with incomplete or unclear data that could not be reliably extracted.

Case reports and case series were included to ensure comprehensive coverage of rare and emerging presentations; however, due to their methodological limitations, they were included only in the qualitative synthesis and excluded from quantitative meta‐analysis.

### 2.3 Data Extraction

Data extraction was performed independently by two reviewers using a standardized data collection form. Extracted information included study characteristics (study design, geographic location, and sample size), participant demographics (age and sex), type of arboviral infection, diagnostic methods (e.g., RT‐PCR, serology, IgM detection), and clinical outcomes such as intensive care unit admission, mechanical ventilation, mortality, and disability.

Additional variables included comorbidities, neurological manifestations, Guillain–Barré syndrome subtypes, and vaccination history where available. Any discrepancies between reviewers were resolved through discussion or, when necessary, consultation with a third reviewer.

### 2.4 Quantitative Synthesis (Meta‐Analysis)

Quantitative synthesis was performed using data from cohort and case–control studies only. For cohort studies, pooled prevalence estimates were calculated to assess the occurrence of Guillain–Barré syndrome among patients with arboviral infections, as well as the prevalence of arboviral infections among patients with Guillain–Barré syndrome.

For case–control studies, pooled odds ratios (ORs) were estimated to evaluate the association between Guillain–Barré syndrome and specific arboviruses, including Zika, chikungunya, and Japanese encephalitis viruses.

Random‐effects models were applied to account for between‐study variability. Where sufficient data were available, subgroup analyses were conducted according to arbovirus type. Case reports and case series were not included in any quantitative analyses.

### 2.5 Risk of Bias and Quality Assessment

The methodological quality of included studies was assessed using design‐specific critical appraisal tools. The Joanna Briggs Institute (JBI) checklist for prevalence studies was applied to relevant studies, while the JBI checklist for case–control studies was used for analytical designs [14].

All studies were independently evaluated by two reviewers, with disagreements resolved through discussion. Case reports and case series were not subjected to formal risk‐of‐bias assessment due to their descriptive nature and were included solely in the qualitative synthesis.

### 2.6 Statistical Analysis

All statistical analyses were conducted using R software (packages *meta* and *metafor*). Pooled estimates were calculated using random‐effects models to account for anticipated heterogeneity across studies. Heterogeneity was assessed using the I^2^ statistic, with values greater than 50% considered indicative of substantial heterogeneity.

Pooled effect sizes are presented with 95% confidence intervals (CIs), and prediction intervals were calculated where appropriate to reflect between‐study variability. For prevalence analyses, logit transformations were applied and subsequently back transformed to proportions to enhance interpretability.

Forest plots were generated to visualize individual and pooled estimates, while funnel plots were used to assess potential publication bias.

## 3 Results

### 3.1 Study Selection, Characteristics, and Participants

**Figure 1:**
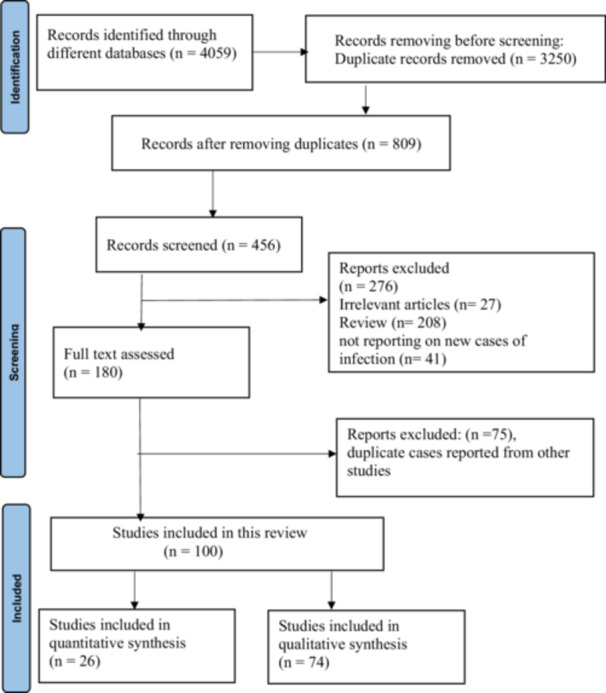
PRISMA flow diagram illustrating the study selection process for the systematic review and meta‐analysis of arbovirus‐associated Guillain–Barré syndrome (GBS), including identification, screening, eligibility assessment, and final study inclusion.

A total of 4059 studies were identified through database searches, of which 3,250 were duplicates. Following the removal of duplicates and screening of abstracts and full texts against predefined inclusion criteria, 100 studies were included in the final analysis [15, 16, 17, 18, 19, 20, 21, 22, 23, 24, 25, 26, 27, 28, 29, 30, 31, 32, 33, 34, 35, 36, 37, 38, 39, 40, 41, 42, 43, 44, 45, 46, 47, 48, 49, 50, 51, 52, 53, 54, 55, 56, 57, 58, 59, 60, 61, 62, 63, 64, 65, 66, 67, 68, 69, 70, 71, 72, 73, 74, 75, 76, 77, 78, 79, 80, 81, 82, 83, 84, 85, 86, 87, 88, 89, 90, 91, 92, 93, 94, 95, 96, 97, 98, 99, 100, 101, 102, 103, 104, 105, 106, 107, 108, 109, 110, 111, 112, 113]. These comprised 74 case reports/case series [15, 16, 17, 18, 19, 20, 21, 22, 23, 24, 25, 26, 27, 28, 29, 30, 31, 32, 33, 34, 35, 36, 37, 38, 39, 40, 41, 42, 43, 44, 45, 46, 47, 48, 49, 50, 51, 52, 53, 54, 55, 56, 57, 58, 59, 60, 61, 62, 63, 64, 65, 66, 67, 68, 69, 70, 71, 72, 73, 74, 75, 76, 77, 78, 79, 80, 81, 82, 83, 84, 85, 86, 87], 19 prevalence studies [88, 89, 90, 91, 92, 93, 94, 95, 96, 97, 98, 99, 100, 101, 102, 103, 104, 105, 106], and seven case‐control studies [107, 108, 109, 110, 111, 112, 113]. Participants were diagnosed with either Guillain‐Barré syndrome (GBS) or one of six arboviral infections: DENV, ZIKV, CHIKV, WNV, JEV, or TBEV, with some cases involving co‐infections. Data extracted included study characteristics, participant demographics, clinical outcomes, comorbidities, and vaccination history. The PRISMA flow diagram is presented in Figure 1.

### 3.2 Geographic Distribution

Cohort studies (*n* = 19) covered 27 countries and regions, with distribution as follows: North America (37%), South America (26%), Asia (21%), Africa (1%), and Oceania (0.05%) (Table 1). Case‐control studies (*n* = 7) were conducted in North America (*n* = 3), Asia (*n* = 2), and Oceania (*n* = 2) (Table 2).

**Table 1:** General study characteristics of prevalence studies.

Study name	Study location	Sample size/Virus examined	Diagnostic methods	Study population
Economopoulou (2009)	Réunion France	610 CHIKV	IgM/RT PCR	610 total severe CHIKV cases
Lemant (2008)	Réunion France	19 CHIKV	Serum, CSF. IgM/RT PCR	ICU with acute CHIKV
Bonifay (2018)	French Guiana	96 CHIKV	Serum. IgM/PRNT	Nonpregnant adults with CHIKV
Thiery (2015)	Guadeloupe, Martinique	17 CHIKV	Acute CHIKV infection not specified	Critically ill patients with Chikungunya virus infection
Oehler (2015)	France	50 CHIKV	Serum. RT PCR/IgM	CHIKV patients with complications
Tun (2020)	Sri lanka	295 DENV	Serum. IgM/RT PCR/NS1	Severe DENV manifestations during the outbreak, 2017
Sejvar (2005)	Northern Colorado/US	32 WNV	Serum. IgM/PRNT	patients with paralysis and acute WNV infection
Wang (2020)	China	161 JEV, WNV, DENV	Serum, CSF. IgM/RT PCR	161 patients with confirmed JEV infection
Sebastián (2017)	8 countries. Latin America	10, ZIKV/DENV	serum, CSF, urine. IgM/RT PCR	16 ICUs in 8 countries enrolled 49 critically ill patients.
Zambrano (2019)	Honduras	108, ZIKV/DENV/CHIKV	Serum. RT PCR/IgM	patients met the diagnostic criteria for GBS, according to the Brighton Collaboration (levels 1 or 2)
Baskar (2018)	India (Southern)	90, ZIKV/DENV/JEV	Serum. RT PCR/IgM	GBS cases, age more than 12 years
Del Carpio‐Orantes (2020)	Mexico	34, ZIKV/DENV/CHIKV	Serum, urine, CSF. RT PCR/IgM	Brighton criteria 1‐3, with serological tests for arboviruses
Parra (2016)	Colombia	42, ZIKV/DENV	Serum, urine, CSF. RT PCR/IgM	Patients with Brighton criteria for GBS
Rozé (2017)	Martinique/French West Indies	30, ZIKV/DENV	Serum, urine, CSF. RT PCR/IgM/NT	data from GBS meeting levels 1 or 2 the Brighton Collaboration, with proof of recent ZIKV
Dirlikov (2016)	Puerto Rico/US	56, ZIKA/flavivirus	Serum, urine, saliva, CSF. RT PCR/IgM	Brighton Collaboration criteria level 1, GBS
Balavoine (2017)	West Indies/France	27, CHIKV	Serum, CSF. RT PCR/IgM	All GBS cases diagnosed during the 2014 outbreak
Matos (2020)	Brazil	34, ZIKV/DENV/CHIKV	Serum, CSF. RT PCR/IgM	patients aged 15 year or older with the diagnosis of GBS (Brighton criteria I or II).
Ravi (1994)	India (Bangalore)	34, JEV/DENV/WNV	Serum, CSF. Isolation using Aedes albopictus/IgM	cases of GBS
Leonhard (2021)	Brazil	71, ZIKV/DENV/CHIKV	Serum, CSF. RT PCR/IgM	GBS patients from a regional neurology center in Northeast Brazil

**Table 2:** Summary of general characteristics of case control studies.

Study name	Study location	Sample size/Virus examined	Diagnostic methods	Study population
Smith (2018)	New Caledonia	7, 30. ZIKV	Serum. IgM	patients with GBS level 1 or 2 according to Brighton criteria, control, patients consulting for non‐febrile illness at Institute Pasteur de Nouvelle‐Calédonie.
Grijalva (2020)	Mexico	97, 184. ZIKV/DENV	Serum, urine. IgM/RT PCR	GBS patients, Controls, non‐febrile disease
Dirlikov (2017)	Puerto Rico/US	39,39. ZIKV	Serum, urine. IgM/RT PCR	GBS level1, Case‐patients were matched to community controls 1:2 by age group and place of residence.
GeurtsvanKessel (2018)	French Polynesia	42,98. ZIKV	Serum. IgM/neuterlizing Ab	GBS patients, controls were non‐febrile age‐matched, sex‐matched, and residence‐matched patients who presented at the hospital
Cao‐Lormeau (2016)	Bangladesh	418,418. ZIKV	Serum. RT PCR/IgM	GBS patient defined by NINDS. Control healthy family member
Stegmann‐Planchard (2020)	French West Indies	24/72. CHIKV	Serum. RT PCR/IgM	GBS during the 2014 outbreak in the French West Indies and controls were blood donors during the same period
Dutta (2021)	India	150,150. CHIKV/JEV	Serum. IgM	150 treatment‐naive patients with GBS and 150 age and sex‐matched controls

Among case reports and case series (*n* = 74), 17 studies (23%) originated from South America, 28 (38%) from Asia, 20 (27%) from North America, 2 (3%) from Europe, 4 (5%) from Oceania, and 3 (4%) from Africa. The viral infections examined included ZIKV (27 studies), CHIKV (8), DENV (25), WNV (8), JEV (5), and TBEV (1). ZIKV studies primarily focused on Latin America or individuals with travel history to Latin America during the 2015–2016 outbreaks. DENV cases were distributed across Asia (India, Sri Lanka, Pakistan, Taiwan, Malaysia, Nepal, Bangladesh), South America (mainly Brazil), and other regions, including the Caribbean, Africa, and Oceania (1989–2024). CHIKV cases were reported in Asia (India, Pakistan), Africa (Réunion), South America (Colombia, Paraguay, Brazil), and Oceania (French Polynesia), with study periods from 2006 to 2023. WNV cases were mainly reported in North America (primarily the US) and Israel (1999–2024). JEV cases were observed in North America (US) and Asia (China, India), and a single TBEV case was reported from Germany in 2019. General study characteristics are summarized in Table 3 and Tables S1–S4.

**Table 3:** Summary of general characteristics of case reports/case series.

Region	Number of studies (%)	Viral infections examined	Study locations
South America	17 (23)	ZIKV, DENV, CHIKV	ZIKV, CHIKV, DENV primarily in Brazil, with some in other South American countries.
Asia	28 (38)	ZIKV, DENV, CHIKV, JEV	DENV in India, Sri Lanka, Pakistan, Taiwan, Malaysia, Nepal, Bangladesh; CHIKV in India and Pakistan; JEV in China and India.
North America	20 (27)	ZIKV, DENV, WNV, JEV	DENV in the US and other regions; WNV in the United States and Israel; JEV in the US.
Europe	2 (3)	CHIKV, WNV, JEV	CHIKV in Netherlands, TBEV in Germany
Oceania	4 (5)	ZIKV, CHIKV, DENV	ZIKV cases linked to travel in French Polynesia, DENV in New Caledonia, CHIKV in French Polynesia.
Africa	3 (4)	DENV, CHIKV	DENV (Sudan), CHIKV (Réunion)

### 3.3 Meta‐Analysis for Prevalence Studies

Nineteen prevalence studies investigated the bidirectional association between GBS and arboviruses, evaluating both the risk of GBS following arboviral infection and the prevalence of arboviral infection among GBS cases. Nine studies assessed GBS prevalence among patients with confirmed arbovirus infection [89, 90, 91, 92, 93, 94, 95, 96, 97]. Among these, three studies focused on a primary virus while also testing for additional arboviruses: CHIKV (also testing for DENV and ZIKV), JEV (also testing for WNV and DENV), and ZIKV (also testing for DENV). The remaining six studies examined GBS incidence in patients with a single virus—four on CHIKV, one on DENV, and one on WNV.

**Figure 2:**
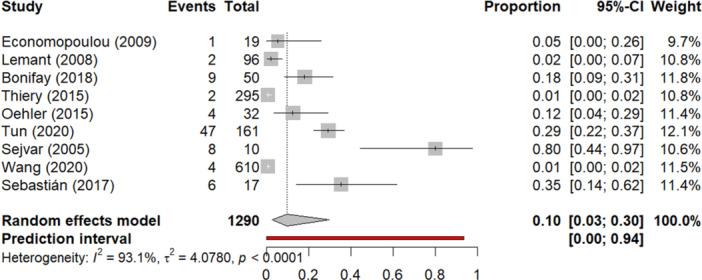
Forest plot showing the pooled prevalence of Guillain–Barré syndrome among patients with laboratory‐confirmed arboviral infections.

**Figure 3:**
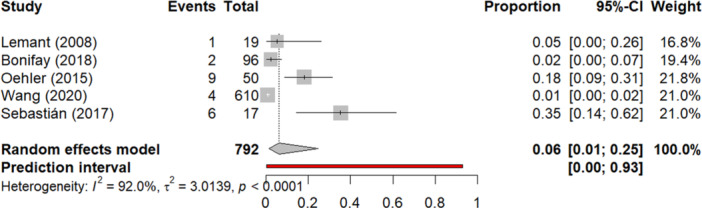
Forest plot of subgroup analysis showing the pooled prevalence of Guillain–Barré syndrome among patients with chikungunya virus (CHIKV) infection.

The pooled prevalence of GBS among patients with arboviral infection was 1% (95% CI: 0.3%–3.3%). Funnel plot regression analysis revealed no significant publication bias (*p* = 0.18; Figure S1). Virus‐specific prevalence ranged from 0.7% for DENV (2/295) to 80% for ZIKV (8/10), 13% for WNV (4/32), and 29% for JEV (47/161). A subgroup analysis for CHIKV (five studies) estimated a pooled prevalence of 6% (95% CI: 0.01–0.25; *I* ^2^ = 92%; *p* < 0.0001). Forest plots are shown in Figures 2 and 3.

**Figure 4:**
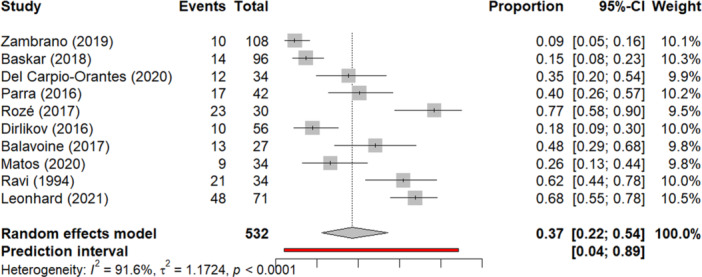
Forest plot showing the pooled prevalence of arboviral infections among patients with Guillain–Barré syndrome.

**Figure 5:**
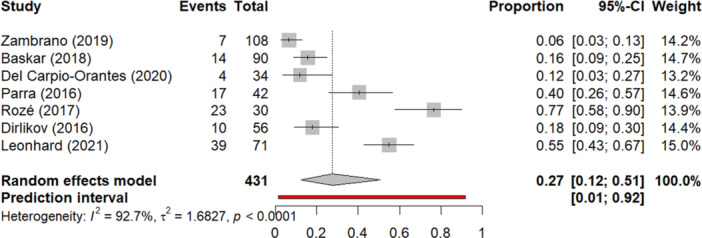
Forest plot of subgroup analysis showing the pooled prevalence of Zika virus (ZIKV) infection among patients with Guillain–Barré syndrome.

**Figure 6:**
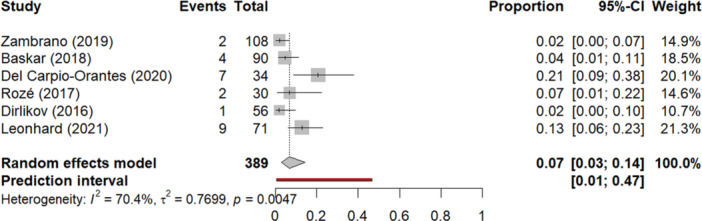
Forest plot of subgroup analysis showing the pooled prevalence of dengue virus (DENV) infection among patients with Guillain–Barré syndrome.

**Figure 7:**
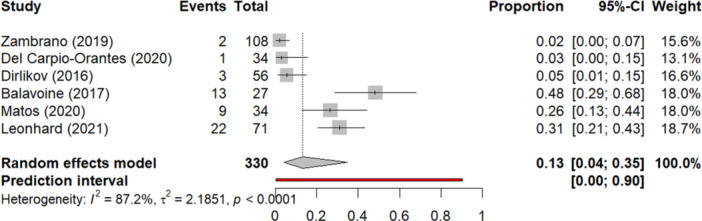
Forest plot of subgroup analysis showing the pooled prevalence of chikungunya virus (CHIKV) infection among patients with Guillain–Barré syndrome.

Ten studies examined the prevalence of arboviral infections among GBS patients [98, 99, 100, 101, 102, 103, 104, 105, 106, 107]. Among these, four included ZIKV, DENV, and CHIKV diagnoses; one utilized a diagnostic panel for ZIKV and flavivirus antibodies; two reported ZIKV and DENV; one examined ZIKV, DENV, and JEV; one reported JEV, WNV, and DENV; and one focused solely on CHIKV. The pooled prevalence of arbovirus infection among GBS patients was 37% (95% CI: 0.22–0.54; I^2^ = 92%; *p* < 0.0001), with no evidence of publication bias (*p* = 0.99; Figure S2). Subgroup meta‐analyses for ZIKV (*n* = 7), DENV (*n* = 6), and CHIKV (*n* = 6) showed pooled prevalences of 27% (95% CI: 0.12–0.51; I^2^ = 93%), 7% (95% CI: 0.03–0.14; *I* ^2^ = 70%), and 13% (95% CI: 0.04–0.35; *I* ^2^ = 87%), respectively (Figures 4, 5, 6, 7).

**Figure 8:**
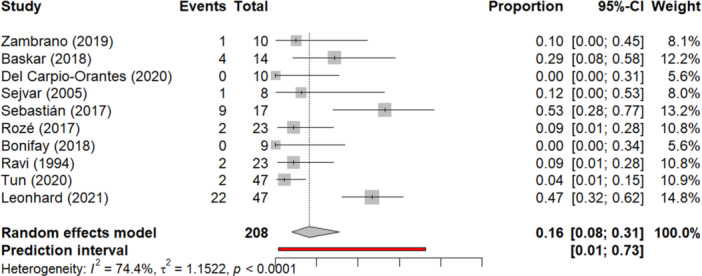
Forest plot showing the pooled prevalence of laboratory‐confirmed arboviral co‐infections among patients with Guillain–Barré syndrome.

Ten studies reported co‐infections among GBS patients confirmed by IgM and/or RT‐PCR. Random‐effects meta‐analysis indicated that 16% of GBS cases (95% CI: 0.08–0.31; *I* ^2^ = 74%; *p* < 0.0001) involved multiple arboviruses (Figure 8, Table 4).

**Table 4:** Co‐infection frequency in GBS cases with recent infection according to IgM/RT‐PCR.

Study	Virus examined	Number of Co‐infections	Details
Zambrano (2019)	ZIKV, DENV	1/10	1 case of ZIKV/DENV co‐infection
Baskar (2018)	ZIKV, DENV	4/14	4 cases of ZIKV/DENV co‐infection
Del Carpio‐Orantes (2020)	ZIKV, DENV, CHIKV	0/10	No co‐infections detected
Sejvar (2005)	ZIKV/DENV	1/8	1 case of ZIKV/DENV co‐infection
Sebastián (2017)	Antiflavivirus (combination of flaviviruses)	9/17	9 cases of flavivirus co‐infection
Rozé (2017)	ZIKV/DENV	2/23	2 cases of ZIKV/DENV co‐infection
Bonifay (2018)	ZIKV, DENV, CHIKV	0/9	No co‐infections detected
Ravi (1994)	JEV, WNV, DENV	2/23	2 cases of JEV/WNV/DENV co‐infection
Tun (2020)	JEV, DENV, WNV	2/47	2 cases of JEV/DENV/WNV co‐infection
Leonhard (2021)	ZIKV, DENV, CHIKV	22/47	14 cases of ZIKV/CHIKV and 8 ZIKV/DENV co‐infection

### 3.4 Meta‐Analysis for Case‐Control Studies

**Figure 9:**
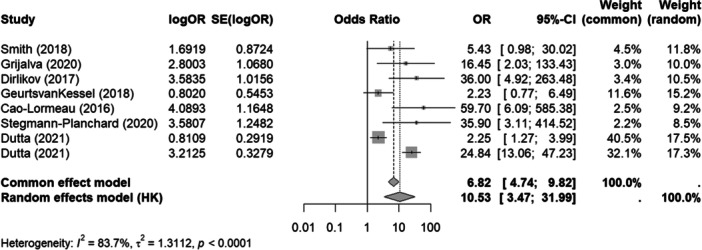
Forest plot showing pooled odds ratios from case‐control studies evaluating the association between arboviral infections and Guillain–Barré syndrome, including subgroup analyses.

Seven case‐control studies were analyzed [108, 109, 110, 111, 112, 113, 114], with four focusing on ZIKV, one on CHIKV, and one reporting separate ORs for CHIKV and JEV. The pooled OR was 10 (95% CI: 3.4–32; *I* ^2^ = 84%; *p* < 0.0001), with no significant publication bias (*p* = 0.37; Supplementary Figure S3). Subgroup analyses showed ORs of 8 for ZIKV (95% CI: 2–34; I^2^ = 58%; *p* = 0.07) and 5 for CHIKV (95% CI: 0–40; *I* ^2^ = 55%; *p* = 0.14), suggesting a strong association for ZIKV but limited evidence for CHIKV. Forest plots are presented in Figure 9.

### 3.5 Clinical Outcomes

Pooled analysis of 11 cohort studies (*n* = 213) indicated an ICU/mechanical ventilation (MV) admission rate of 53% (95% CI: 0.31–0.73; *I* ^2^ = 70%; *p* < 0.0002), with subgroup rates of 40% for CHIKV (*n* = 4; 95% CI: 0.24–0.56; *I* ^2^ = 0%) and 44% for ZIKV (n = 3; 95% CI: 0.095–0.86; I^2^ = 77%; *p* < 0.0001). Mortality was 15% overall (95% CI: 0.07–0.30; I^2^ = 64%; *p* = 0.01), highest in JEV (21 cases), followed by ZIKV (4) and CHIKV (2). Disability (*n* = 6) occurred in 51% (95% CI: 0.36–0.64; *I* ^2^ = 11%; *p* = 0.34), with 54% in CHIKV cases (*n* = 5). Functional recovery was observed in 25% (95% CI: 0.12–0.42; *I* ^2^ = 50%; *p* = 0.03), including 30% for CHIKV and 29% for ZIKV (Table S5).

### 3.6 Clinical Outcomes Across Arboviral Infections

Evidence from case reports and case series (84 patients) demonstrated substantial variability in clinical outcomes across different arboviral infections. Reports involving ZIKV, DENV, CHIKV, WNV, JEV, and TBEV described a broad spectrum of recovery and complication patterns.

ZIKV‐associated cases most frequently reported partial recovery and the need for intensive care or mechanical ventilation, with some cases complicated by neurological sequelae and occasional mortality. DENV‐related cases more often reported favorable recovery outcomes, although disability and ICU admission were also described. CHIKV‐associated cases were characterized by a high frequency of severe disease requiring intensive care and mechanical ventilation, alongside frequent neurological and occasional cardiac complications. WNV and JEV cases showed mixed outcomes, including disability, neurological complications, and mortality. A single TBEV‐associated case reported full recovery without complications (Table 5 and S6).

**Table 5:** Infection‐specific outcomes in case reports and case series studies.

Arbovirus	Full recovery (F/R)	Disability	ICU admissions/MV	Neurological complications	Cardiac issues	Death
ZIKV (*n* = 21)	19.05%	28.57%	28.57%	9.52%	9.52%	9.52%
DENV (*n* = 36)	50.00%	30.56%	22.22%	0.00%	2.78%	5.56%
CHIKV (*n* = 13)	0.00%	46.15%	92.31%	30.77%	7.69%	7.69%
WNV (*n* = 8)	22.22%	44.44%	33.33%	11.11%	11.11%	11.11%
JEV (*n* = 5)	25.00%	25.00%	25.00%	25.00%	25.00%	25.00%
TBEV (*n* = 1)	100.00%	0.00%	0.00%	0.00%	0.00%	0.00%

Overall, these indicates that reported outcomes ranged from full or minor recovery to severe disability and death, highlighting marked heterogeneity in clinical trajectories. Among patients with multiple arboviral infections, most reports described severe outcomes, including prolonged mechanical ventilation and combined neurological and cardiac manifestations, with only a small number achieving full recovery.

### 3.7 Neurological Manifestations, Comorbidities, and Vaccination History

Neurological symptoms typically developed after febrile illness, sometimes concurrently or up to 1 month later, with some cases presenting without prior fever. Common manifestations included progressive limb weakness, paresthesia, areflexia, respiratory failure, bilateral facial palsy, dysphagia, dysphonia, quadriparesis, and autonomic dysfunction.

The mean interval from febrile illness to GBS diagnosis was 9 days for ZIKV, 10 days for DENV, 13 days for CHIKV, 14 days for WNV, and 7 days for JEV.

Comorbidities included hypertension (10), diabetes mellitus (5), pregnancy (2), hyperthyroidism, hypothyroidism, smoking, asthma, Staphylococcus aureus pneumonia, acute articular rheumatism, scoliosis, gastroesophageal reflux, chronic pancreatitis (DENV), and depression with BPH. Vaccination history was reported in a few cases: three JEV‐related GBS following vaccination, one ZIKV‐related case post–Yellow Fever vaccination, and one TBEV‐related case post–TBEV vaccination. Most cases lacked detailed vaccination histories.

### 3.8 Diagnosis, GBS Subtypes, and Treatment

Arboviral involvement was confirmed via serology and molecular diagnostics. ZIKV: 127 tested, 59 IgM‐positive, 8 RT‐PCR only, 60/101 RT‐PCR positive across serum, urine, and CSF. DENV: 48 IgM, NS1 antigen, and RT‐PCR confirmation. CHIKV: 45 IgM, some RT‐PCR. WNV: 10 confirmed, IgM and PRNT. JEV: 70 IgM, majority positive in serum and CSF, confirmed by RT‐PCR (Table S7).

Among 151 cases, GBS subtypes were 50% primary demyelinating, 43% primary axonal, and 7% other variants. CHIKV: 86% demyelinating; ZIKV: 92% demyelinating; DENV: 40% demyelinating, 51% axonal, 5% other variants; WNV: 57.1% demyelinating, 25% axonal, 12.5% MFS; JEV: 18% demyelinating, 78% axonal, 4% MFS/BEE.

Treatment (n = 185) included IVIG only (132), plasma exchange only (8), combined IVIG/PE (18), steroids only (21), no treatment (5), and physiotherapy only (1). Most patients with significant disability were referred to rehabilitation. CSF analysis (n = 133) showed 100 compatibles with albuminocytologic dissociation, 23 with pleocytosis, and 10 normal; 13 of the pleocytosis cases were JEV‐related.

### 3.9 Methodological Quality Assessment

Methodological quality was evaluated using the JBI Critical Appraisal Checklist for Studies Reporting Prevalence Data. Studies were classified as low (80–100%), moderate (50–80%), or high (20–50%) risk of bias. Of 26 studies assessed, 13 were low risk, 13 moderate risks, and none high risk. Detailed assessments are presented in Table S8.

## 4 Discussion

This systematic review and meta‐analysis highlight the complex and evolving relationship between arboviral infections and Guillain–Barré Syndrome (GBS) across diverse geographic regions and populations. The global expansion of arboviruses such as ZIKV, DENV, and CHIKV, driven by factors including climate change, urbanization, and increased international travel, underscores the importance of understanding their potential neurological complications [4, 6].

Among the studied arboviruses, ZIKV demonstrates the most consistent association with GBS, particularly during the 2015–2016 epidemic in the Americas, where temporal correlations between viral outbreaks and increased GBS incidence were observed [6]. In endemic regions of Asia and other parts of the world, DENV and CHIKV also appear to contribute to GBS burden, reflecting local epidemiological patterns. However, the distribution of reported cases likely reflects differences in surveillance capacity and diagnostic infrastructure, with potential under‐recognition in resource‐limited settings, particularly in parts of Africa where arboviruses are endemic and diagnostic facilities remain limited [114].

Variability in GBS subtypes associated with different arboviruses may provide insights into underlying pathophysiological mechanisms. ZIKV‐associated cases are more frequently linked to demyelinating forms, supporting an immune‐mediated process, potentially involving molecular mimicry and cross‐reactive antibodies targeting peripheral nerve components [115, 116]. In contrast, DENV and CHIKV exhibit a broader spectrum of GBS subtypes, including axonal variants, which may reflect heterogeneous immune responses or differing host–pathogen interactions [115]. Similarly, findings from Japanese encephalitis virus (JEV)–associated cases suggest possible neurotropic mechanisms; however, direct neuroinvasion cannot be consistently established based on the available evidence [117, 118].

Cerebrospinal fluid (CSF) findings in most cases were consistent with albumin–cytologic dissociation (ACD), characterized by elevated protein levels without significant pleocytosis, in line with previous reports [119]. Nonetheless, a subset of JEV‐associated cases demonstrated pleocytosis, which may indicate a more pronounced inflammatory response or overlap with central nervous system involvement, although such findings are not specific and should be interpreted with caution [120, 121, 122].

Management strategies for arbovirus‐associated GBS remain largely aligned with standard GBS treatment approaches, including intravenous immunoglobulin (IVIG) and plasma exchange (PE), with some cases receiving corticosteroids in combination [123]. Variations in treatment approaches across studies likely reflect differences in disease severity and local clinical practices rather than virus‐specific protocols. A substantial proportion of patients required intensive care support, including mechanical ventilation, and many experienced prolonged recovery or residual disability, suggesting a potentially severe clinical course in some cases. Mortality rates varied across studies, with higher mortality reported in some JEV‐associated cohorts, potentially reflecting more severe inflammatory responses [84, 121].

The pooled prevalence estimates indicated that approximately 1% of individuals with arboviral infections developed GBS, while arboviral infections were identified in a notable proportion of GBS cases. Subgroup analyses suggested variability across viruses, with higher proportions observed for ZIKV compared to DENV and CHIKV. However, these estimates should be interpreted cautiously due to substantial heterogeneity in study design, population characteristics, and diagnostic approaches, as well as differences between outbreak‐based and endemic settings.

Diagnostic confirmation of arboviral infection in the context of GBS remains challenging. In most studies, diagnosis relied on serological testing, particularly IgM detection in serum or cerebrospinal fluid, as GBS typically develops after the acute viremic phase has resolved. While molecular assays represent the gold standard for detecting acute infection, their utility is limited in this context due to transient viremia. Consequently, reliance on serological methods introduces the potential for misclassification due to antibody persistence and cross‐reactivity, particularly in regions with co‐circulating arboviruses. ZIKV detection often required plaque reduction neutralization testing (PRNT) and urine RT‐PCR, with some cases negative in serum or CSF but positive in urine or serology, emphasizing the importance of multimodal diagnostic approaches [124]. Occasional reports of viral detection in blood or CSF raise the possibility of neurotropic involvement, although such evidence remains limited and inconclusive.

Data on vaccination history were limited, with only isolated reports of GBS following vaccination against TBEV or yellow fever virus. Current evidence does not support a definitive causal relationship, underscoring the need for further systematic evaluation of vaccine‐related GBS risk [125, 126].

Overall, this study synthesizes a fragmented and heterogeneous body of evidence to provide a comprehensive overview of arbovirus‐associated GBS. The findings highlight a growing body of evidence supporting an association between arboviral infections and GBS, while emphasizing variability in clinical presentation, diagnostic challenges, and outcomes. Future research should prioritize well‐designed prospective and multicenter studies, standardized diagnostic protocols, and deeper investigation into immune‐mediated and potential neurotropic mechanisms. Strengthening surveillance systems, particularly in resource‐limited settings, will also be critical to better define the global burden and inform clinical and public health responses.

### 4.1 Limitations

This study has several important limitations. The synthesis of clinical manifestations was based on predominantly case reports and case series, limiting causal inference and generalizability, although it provides a comprehensive overview of severe neurological presentations in arbovirus‐related Guillain–Barré syndrome (GBS). The meta‐analysis included a limited number of heterogeneous observational studies with variability in design, diagnostics, and settings, which may have introduced residual confounding. As a systematic review, inclusion was restricted to available evidence, and higher‐quality study designs such as randomized controlled trials were not consistently available. Arboviral diagnosis was largely based on serological testing, as viremia is often transient and resolves before GBS onset, limiting molecular confirmation and definitive evidence of central nervous system involvement, and introducing potential misclassification. Subgroup analyses, including geographical stratification of GBS subtypes, were limited by inconsistent reporting across studies. In addition, potential publication bias and the predominance of studies from middle‐ and high‐income countries may affect generalizability to low‐income settings. This systematic review was not prospectively registered before study initiation. However, the review methodology and eligibility criteria were predefined prior to study selection and data extraction, and the study was conducted in accordance with established systematic review guidelines. Nevertheless, the lack of prospective registration may increase the risk of reporting bias and should be considered when interpreting the findings. Finally, inconsistent reporting of key variables such as comorbidities, vaccination history, and long‐term outcomes limited further analysis of prognostic factors.

## Author Contributions

**Eman Taha Osman Ali:** conceptualization, methodology, data curation, supervision, formal analysis, validation, investigation, visualization, writing – review and editing, writing – original draft, software. **Pierre Gashema:** writing – original draft, methodology, conceptualization, writing – review and editing. **Patrick Gad Iradukunda:** conceptualization, methodology, validation, writing – review and editing. **Emmanuel Edwar Siddig:** conceptualization, methodology, writing – review and editing. **Claude Mambo Muvunyi:** conceptualization, methodology, data curation, supervision, validation, investigation, visualization, project administration, writing – review and editing.

## Funding

The authors have nothing to report.

## Conflicts of Interest

The authors declare no conflicts of interest.

## Supporting information


**Figure S1:** Funnel plot assessing publication bias in prevalence studies evaluating Guillain–Barré syndrome among arbovirus‐infected patients.

**Figure S2:** Funnel plot assessing publication bias in studies evaluating the prevalence of arboviral infections among patients with Guillain–Barré syndrome.

**Figure S3:** Funnel plot assessing publication bias in case‐control studies estimating the association between arboviral infections and Guillain–Barré syndrome.

**Table S1:** General characteristics of included cohort studies.
**Table S2:** General characteristics of included case‐control studies.
**Table S3:** General characteristics of included case reports.
**Table S4:** General characteristics of included case series.
**Table S5**: Pooled meta‐analysis of ICU/MV admission, mortality, disability, and functional recovery rates in arbovirus‐associated GBS (cohort studies).
**Table S6:** Summary of clinical outcomes (full recovery, disability, ICU/MV, neurological and cardiac complications, mortality) from case reports and case series.
**Table S7:** Frequency and types of arbovirus co‐infections among GBS patients (laboratory‐confirmed cases).**Table S8:** Risk of bias assessment for prevalence studies using the Joanna Briggs Institute (JBI) Critical Appraisal Checklist (includes individual study scores and risk category).

**Table S9:** PRISMA 2020 checklist for systematic reviews and meta‐analyses.
**Table S10:** Keywords and search strategy used for literature identification.

## Data Availability

The data that support the findings of this study are openly available in Figshare at https://figshare.com/10.6084/m9.figshare.30252538.
