# Supplementary figures and images for: Arbovirus‐Associated Guillain–Barré Syndrome: A Systematic Review and Meta‐Analysis of Clinical Characteristics, Subtypes, and Vaccine Associations
Source: Immun Inflamm Dis. 2026 Jul 6;14(7):e70483. doi: 10.1002/iid3.70483 (PMC13338631; doi:10.1002/iid3.70483)

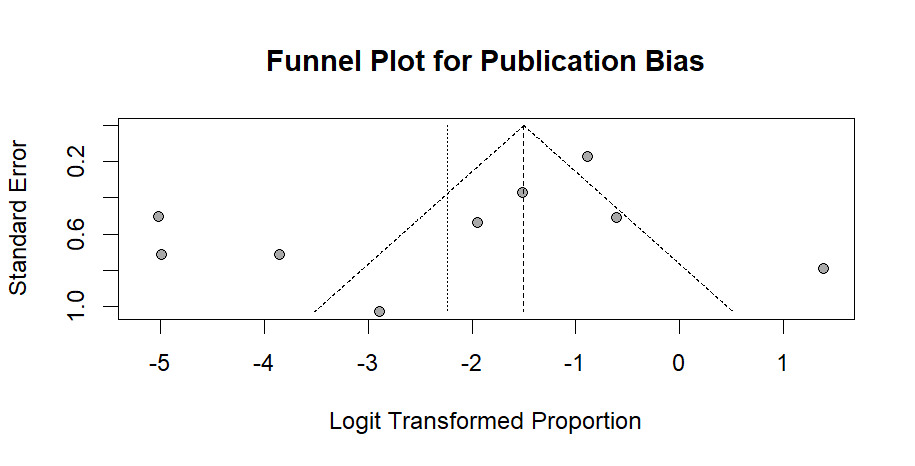

Supplement: Supplementary file 1 — Figure S1: Funnel plot assessing publication bias in prevalence studies evaluating Guillain–Barré syndrome among arbovirus‐infected patients. [file IID3-14-e70483-s002.png]

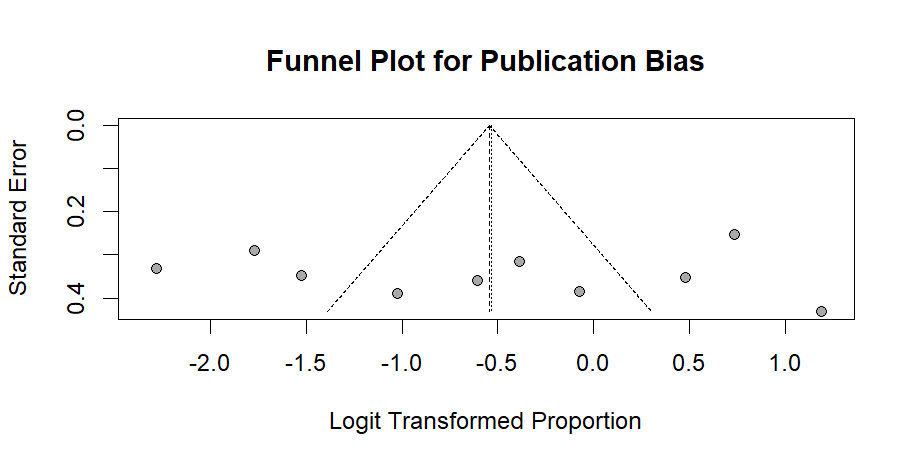

Supplement: Supplementary file 2 — Figure S2: Funnel plot assessing publication bias in studies evaluating the prevalence of arboviral infections among patients with Guillain–Barré syndrome. [file IID3-14-e70483-s004.png]

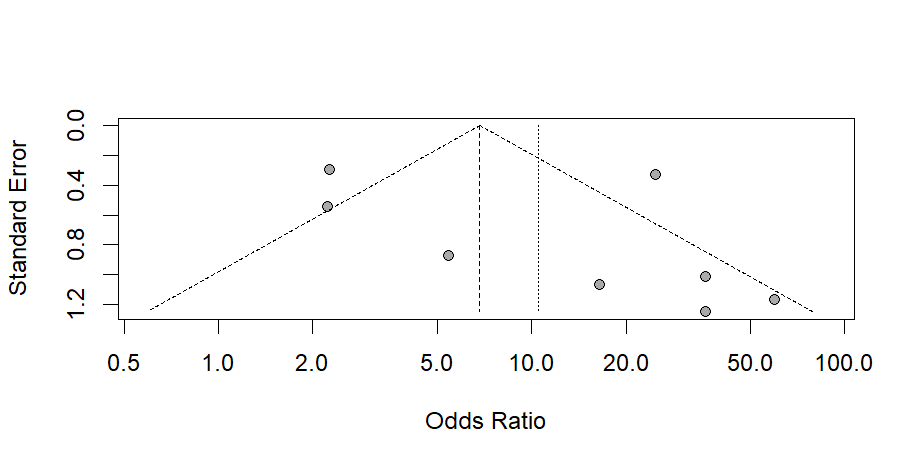

Supplement: Supplementary file 3 — Figure S3: Funnel plot assessing publication bias in case‐control studies estimating the association between arboviral infections and Guillain–Barré syndrome. [file IID3-14-e70483-s001.png]
