# Supplementary material for: Arbovirus‐Associated Guillain–Barré Syndrome: A Systematic Review and Meta‐Analysis of Clinical Characteristics, Subtypes, and Vaccine Associations
Source: Immun Inflamm Dis. 2026 Jul 6;14(7):e70483. doi: 10.1002/iid3.70483 (PMC13338631; doi:10.1002/iid3.70483)
Supplement: Supplementary file 4 — Table S1: General characteristics of included cohort studies. Table S2: General characteristics of included case‐control studies. Table S3: General characteristics of included case reports. Table S4: General characteristics of included case series. Table S5: Pooled meta‐analysis of ICU/MV admission, mortality, disability, and functional recovery rates in arbovirus‐associated GBS (cohort studies). Table S6: Summary of clinical outcomes (full recovery, disability, ICU/MV, neurological and cardiac complications, mortality) from case reports and case series. Table S7: Frequency and types of arbovirus co‐infections among GBS patients (laboratory‐confirmed cases).Table S8: Risk of bias assessment for prevalence studies using the Joanna Briggs Institute (JBI) Critical Appraisal Checklist (includes individual study scores and risk category). [file IID3-14-e70483-s003.docx]

**Table S 1:** Summary for ZIKV case reports/case series

| Year | Country | Initial Neurological Manifestation | Onset Patterns | Recovery Duration | Clinical Outcomes | Age (Years) | Gender |
| --- | --- | --- | --- | --- | --- | --- | --- |
| 2016 | Brazil | Bilateral peripheral facial diplegia, paresis of lower limbs, difficulty walking | Concurrent onset | 5 days | F/R | 51 | F |
| 2016 | Trinidad and Tobago | Tingling and weakness of lower limbs | Concurrent onset | 10 months | mild persistence of weakness | 29 | M |
| 2018 | New York City/US | Paresthesias in hands and feet, weakness in legs, difficulty walking | Concurrent onset | 4 months | persistent respiratory failure and weakness | 64 | F |
| 2016 | US | NA | NA | NA | Massive subarachnoid hemorrhage, coma | 81 | M |
| 2016 | New Zealand | Progressive limb weakness, numbness, unsteady gait, and dyspnea | Concurrent onset | 6 days | 33 days, persistent limb weakness | 47 | M |
| 2015 | Brazil | Tetraparesis, bifacial palsy, paresthesias, and other sensory disturbances | Concurrent onset | 8 days | 47 days, mild/moderate right facial palsy | 22 | M |
| 2016 | Ecuador | Paresthesia, quadriparesis, facial paralysis | Concurrent onset | 6 days | 12 months, residual motor dysfunction, gait disturbance | 57 | F |
| 2018 | US | Paresthesia, weakness, dysautonomia | Concurrent onset | NA | F/R | 37 | M |
| 2013 | French Polynesia | Paresthesia, muscle weakness, dysautonomia, facial palsy | Concurrent onset | 7 days | 40 days, muscular strength score 85/100 | Early 40s | F |
| 2015 | Netherlands | Muscle weakness, sensory disturbances, limbs hyporeflexia, and facial diplegia | 1-7 days after travel | NA | Respiratory insufficiency, mechanical ventilation | 60 | F |
| 2014 | Colombia | Urinary retention and bilateral lower limb weakness with foot | Concurrent onset | 1 year | 4 months, persistent flaccid paraplegia | 24 | F |
| 2016 | Martinique/France | Numbness in extremities, constipation, gait disturbance | Antecedent illness denied | 10 days | Rehabilitation, improved walking | 50s | NA |
| 2016 | Brazil | Weakness in lower limbs, inability to ambulate | 1 month | 1 year | Residual movement problems | 28 | F |
| 2016 | Canada | Muscle fatigue, arthralgia | 10 days | 2 years | Residual left hip flexor weakness, neuropathic pain | 34 | F |
| 2016 | Brazil | Lower extremity paresthesia, muscle weakness | Antecedent illness denied | 4 months | F/R | 9 | F |
| 2016 | Venezuela | Ascending limb weakness, speech and swallowing difficulties | 10 days | 3 weeks | Hospitalized 39 weeks, vaginal delivery | 28 | F |
| 2016 | Haiti | Bifacial weakness, acral paresthesias | Several days | 3 weeks | Minimal improvement in facial weakness | 35 | M |
| 2016 | Suriname | Muscle weakness, paresthesias in hands and feet | 7 days | 12 weeks | F/R | 40s | M |
| 2020 | Brazil | Ascending paresthesia in the feet | 8 days | 6 months | Long-term sequelae, including ambulation disability | 29 | M |
| 2017 | Brazil | Ascending paresthesia, difficulty walking and speaking, respiratory distress | 2 weeks | 71 days | Respiratory issues, mechanical ventilation | 33 | M |
| 2017 | Thailand | Ascending limb weakness, dysphagia, and difficulty in ambulation | Concurrent onset | 4 weeks | F/R | 36 | F |
| 2016 | Puerto Rico | Progressive weakness in limbs and dysphagia | 10 days | 3 weeks | F/R | 38 | F |
| 2015 | Colombia | Weakness, fatigue, difficulty walking | Concurrent onset | 2 months | Mild weakness of lower extremities | 42 | M |
| 2016 | Brazil | Facial diplegia, paresis of lower limbs | Concurrent onset | 3 months | Improvement in gait and muscle strength | 35 | F |
| 2017 | Thailand | Ascending paralysis, difficulty in swallowing and speaking, respiratory distress | Concurrent onset | 2 weeks | F/R | 26 | F |

**Table S 2:** Summary for DENV case reports/case series

| Year | Country | Initial Neurological Manifestation | Onset Patterns | Recovery Duration | Clinical Outcomes | Age (Years) | Gender |
| --- | --- | --- | --- | --- | --- | --- | --- |
| 2004 | Brazil | Muscle weakness, tetraplegia with areflexia, and respiratory insufficiency | 7 days | 9 weeks | Wheelchair, Walk with assistance | 45 | F |
| 2005 | India | Weakness of all four limbs, areflexia | 7 days | 1 month | F/R | 40 | M |
| 2007 | India | Weakness of lower limbs | 4 days | 15 days | F/R | 2 | M |
| 1999 | Trinidad & Tobago | Flaccid areflexic quadriparesis | 2 weeks | NA | NA | 44 | F |
| 2007 | Taiwan | Generalized weakness and myalgia | 10 days | 7 days | F/R | 73 | F |
| 2004 | India | Weakness of lower limbs | 2 weeks | 3 weeks | F/R | 2.6 | F |
| 2004 | India | Alteration of voice, difficulty swallowing, unable to lift hands | 10 days | NA | F/R | 8 | F |
| 2004 | India | Inability to stand | 2 weeks | 4 weeks | F/R | 1.6 | F |
| 2023 | Malaysia | Bilateral lower limb weakness and areflexia | 2 days | 7 days | F/R, Improvement without immunotherapy | 49 | M |
| 2015 | India | Weakness of all four limbs, dysphagia, and respiratory muscle involvement | 1 week | 4 weeks | Died, no response, ventilator associated | 60 | F |
| 2017 | India | Limb weakness, drooling, inability to chew, decreased speech output | 7 days | 2 weeks | P/R | 48 | M |
| 2019 | India | Dysphagia, nasal regurgitation, bilateral facial weakness | 7 days | 1 week | F/R | 20 | F |
| 2016 | India | Limb weakness with areflexia | 6 days | 16 days | F/R | 60 | M |
| 2015 | Sri Lanka | Weakness of bilateral lower limbs | 2 weeks | 1 month | F/R | 34 | M |
| 2017 | Pakistan | Pain and weakness in both lower limbs | 12 days | NA | P/R | 35 | M |
| 2017 | Pakistan | Lower back ache and weakness in the lower limbs | 5 days | NA | P/R | 69 | M |
| 2017 | Pakistan | Bilateral upper and lower limb weakness | 9 days | NA | P/R | 21 | M |
| 2017 | Pakistan | Ascending weakness | NA | NA | Death | 46 | M |
| 2024 | Sudan | Ascending muscle weakness, areflexia, and numbness in both legs | 10 days | 3 months | Mild residual weakness in lower limbs | 32 | M |
| 2024 | Nepal | Distal weakness in all four limbs | 3 days | NA | Prolonged weakness | 30 | M |
| 2018 | India | Weakness involving both upper and lower limbs | 1 week | A few days | P/R, Walk with assistance | 18 | M |
| 2018 | India | Weakness involving both upper and lower limbs | 1 week | 2 weeks | P/R, Walk with assistance | 15 | M |
| 2011 | Brazil | Dysphagia, dysphonia, vomiting, ascending paresthesia | 10 days | 6 months | P/R, Dysphonia, Gait disturbance | 6 | F |
| 2021 | Pakistan | Swallowing difficulty, bilateral arm and neck weakness | 3 weeks | 4 months | P/R, Rehabilitation therapy | 31 | F |
| 2018 | Sri Lanka | Numbness and pain of the bilateral upper limbs and lower limbs | 2 days | 1 week | P/R | 60 | M |
| 2012 | Bangladesh | Acute flaccid weakness in both upper and lower limbs | 7 days | 6 weeks | P/R | 39 | F |
| 2012 | New Caledonia | Tetraparesis with distal predominance | 1 week | NA | P/R | 68 | M |
| 2012 | New Caledonia | Tetraparesis with upper limb and proximal predominance | 1 week | NA | P/R | 55 | F |
| 2012 | New Caledonia | Tetraparesis with upper limb and proximal predominance | 1 week | NA | P/R | 58 | M |
| 2023 | India | Loss of power in both lower limbs | 3 days | 21 days | S/R | 8 | M |
| 2021 | India | Ascending symmetric weakness of all four limbs | 1 week | 3 days | S/R, Ankle pain | 27 | F |
| 2016 | Brazil | Dysphonia, dysphagia, bilateral facial nerve paralysis, tetraparesis, paresthesia, areflexia | 10 days | 9 days, up to 1 year | F/R |  | M |
| 2009 | Brazil | Ascending paraparesis and limb paresthesias | 5 days | 22 days | F/R | 40 | F |
| 2009 | Brazil | Acute paraparesis, unable to walk | 17 days | NA | NA | 52 | F |
| 2009 | Brazil | Paraparesis with paresthesias of the lower limbs | 20 days | NA | F/R without treatment | 66 | M |
| 2009 | Brazil | Acute paraparesis and lumbar pain | 5 days | NA | F/R without treatment | 74 | M |
| 2009 | Brazil | Difficulty walking, ascending paraparesis, areflexia, and paresthesias | NA | 30 days | NA | 51 | F |
| 2020 | India | Weakness in lower limbs | NA | 4 weeks | P/R, 4/5 in left upper limb and 5/5 in right upper limb | 21 | M |

양식의 맨 아래

**Table S 3:** Summary for CHIKV case reports/case series

| Year | Country | Initial Neurological Manifestation | Onset Patterns | Recovery Duration | Clinical Outcomes | Age (Years) | Gender |
| --- | --- | --- | --- | --- | --- | --- | --- |
| 2017 | India | Swelling and weakness of distal upper limbs, neck weakness, and tachypnea | 17 days | 1 month | P/R, 4-/5 power in upper limbs and grade 3/5 power in lower limbs | 18 | M |
| 2017 | India | Numbness involving both soles, flaccid weakness, first involving both lower limbs | 11 days | 1 month | P/R, 4-/5 and 3/5 power in upper and lower limbs | 30 | M |
| 2006 | France | Progressing motor weakness and sensory disturbances developed | 1 month | 2 months | F/R | 51 | F |
| 2006 | France | Weakness with facial diplegia and sensory disturbances developed | 2 weeks | NA | F/R | 48 | F |
| 2014 | Colombia | Motor weakness and sensory disturbances | 1 day | 8 weeks | F/R | 77 | F |
| 2018 | India | Ascending quadriparesis involving both lower limbs, facial deviation, slurring of speech or nasal regurgitation | 10 days | 1 week | No progression | 45 | M |
| 2023 | Paraguay | NA | NA | NA | NA | 62 | M |
| 2015 | India | Respiratory distress with hypoxia | 1 month | 2 weeks | P/R | 21 | F |
| 2014 | Pakistan | Difficulty swallowing and left arm weakness | 1 month | 6 months | F/R, 4+/5 power in left upper limb | 36 | F |
| 2007 | France | Facial diplegia, tingling sensation, weakness | 2 weeks | 1 month | P/R, walk with assistance | 51 | F |
| 2007 | France | Tetraparesis | 3 days | 1 month | P/R, facial paresis | 60 | M |
| 2007 | France | Four limb paresthesia | 1 week | 1 month | P/R, facial diplegia | 49 | F |
| 2019 | Brazil | Lower extremity paresthesia | 1 week | 1 week | NA | 42 | M |

**Table S 4:** Summary for WNV, JEV and TBEV case reports/case series

| Year | Country | Initial Neurological Manifestation | Onset Patterns | Recovery Duration | Clinical Outcomes | Age (Years) | Gender |
| --- | --- | --- | --- | --- | --- | --- | --- |
| 2013 | Aurora/US | Ascending weakness and numbness | 1 month | P/R | NA | 20 | M |
| 1999 | New Orleans, LA/US | Progressive weakness | 1 week | 10 weeks | Nursing home after gastrostomy feeding tube | 69 | M |
| 2020 | New York/US | Weakness and inability to walk | 1 week | 1 month | Lower extremity weakness, 10 days | 65 | F |
| 2011 | Israel | Drowsy and unspecific generalized weakness, high blood pressure | 2 weeks | 7 weeks | F/R | 67 | F |
| 2019 | Omaha, NE/US | Progressive muscle weakness | NA | NA | CANOMAD | 40 | M |
| 2022 | Florida | Bilateral upper and lower extremity weakness, numbness, and tingling | NA | NA | Death, toxic metabolic encephalopathy/pneumonia | 64 | M |
| 2024 | US | Progressive bilateral upper and lower extremity weakness | 6 days | NA | Ptosis, P/R | 49 | M |
| 2024 | US | Bilateral upper and lower extremity weakness | 5 days | 3 weeks | S/R | 54 | M |
| 2023 | US | Altered mental status, diplopia, and ataxia | 6 days | NA | MFS and BBE, bradycardia, mild transaminitis | 27 | M |
| 2014 | China | Generalized weakness, numbness in extremities, bilateral facial nerve paralysis | 5 days | 3 months | F/R | 23 | M |
| 2015 | India | Symmetric quadriparesis with difficulty in rolling over the bed | 3 days | 1 week | Walk with assistance, episodes of GTCS, respiratory distress | 14 | M |
| 2022 | China | Muscle weakness of the bilateral lower limbs with fever | 1 week | 36 days | Death | 18 | M |
| 2022 | China | Fever and disturbance of consciousness | 3 days | 14 months | Brain atrophy, corpus callosum tract partially broken, hemorrhage in the right thalamus | 43 | F |
| 2019 | Germany | Bilateral facial palsy and proximal palsy in the upper right and lower left limb | 12 weeks | 13 weeks | F/R | 65 | M |

**Table S5:** Summary of outcomes and comorbidity for arbovirus related GBS cases

| Study | Virus Type for GBS | Number of GBS Cases | MV/ICU | Death | Follow-Up Duration | Other Disability/Complications | Underlying Conditions |
| --- | --- | --- | --- | --- | --- | --- | --- |
| Lemant (2008) | CHIKV | 1 | 1 | No | 5 months | Moderate flaccid tetraparesis | Diabetes mellitus |
| Bonifay (2018) | CHIKV | 2 | 1 | No | NA | Chronic pancreatitis | Chronic pancreatitis |
| Oehler (2015) | CHIKV, ZIKV, DENV | 9 | 4 (2-15 days) | No | 3 months | Functional rehabilitation | Hypertension, diabetes mellitus |
| Tun (2020) | DENV | 2 | NA | No | 2–10 days | Respiratory difficulty | NA |
| Sejvar (2005) | WNV | 4 | 2 | No | NA | No baseline improvement in strength, 2 cases | NA |
| Wang (2020) | JEV | 47 | 44 | 21 | 8 months | Limb weakness, atrophy, incontinence. | NA |
| Sebastián (2017) | ZIKA, DENV | 8 | NA | 1 | 6 days | Respiratory failure, multisystem organ failure in 1 death | NA |
| Baskar (2018) | ZIKV, DENV, JEV | 14 | 5 | 1 | 3 months | Ventilatory support required, 1 death from pneumonia | NA |
| Rozé (2017) | ZIKA | 23 | 14 | 2 | 5.9 months | 2 deaths from illness | NA |
| Balavoine (2017) | CHIKV | 13 | 5 | 2 | 1 year | 7 good functional recovery, 2 deaths | BPH, Type 2 diabetes, Hypertension, previous DENV infection, depression |
| Matos (2020) | CHIKV | 9 | 2 required MV | No | 6 months | Most recovered, minor residual symptoms | NA |

**Table S6:** Neurological Complications and Cardiac Issues, Case reports/Case series

| Infection | Neurological Complications | Cardiac Issues |
| --- | --- | --- |
| ZIKV | 2 cases:  - transverse myelitis - subarachnoid hemorrhage (coma) | 3 cases:  - Tachycardia |
| DENV | 0 | 1 case:  - Cardiovascular involvement (myocardial infarction) |
| CHIKV | 4 cases:   - 1 Deep coma and encephalitis  -3 Bickerstaff Brainstem Encephalitis (BBE) | 1 case:  - Cardiovascular instability |
| WNV | 1 case:  - chronic ataxic neuropathy | 1 case:  - bradycardia |
| JEV | 1 case:   - Hemorrhage in the right thalamus | 0 |

**Table S7:** Summary of virus diagnosis for arbovirus related GBS cases

| Virus | Number of Cases | IgM Test (Serum) | IgM Test (CSF) | RT-PCR (Serum) | RT-PCR (Urine) | RT-PCR (CSF) | NS1 Detection | PRNT |
| --- | --- | --- | --- | --- | --- | --- | --- | --- |
| ZIKV | 127 | 59 +ve (58 serum, 1 serum + CSF) | 5 +ve (serum + CSF) | 60/101 +ve (25 serum, 34 urine, 1 CSF, 3 both CSF and urine) | - | - | - | - |
| DENV | 48 | 48 +ve | 5 +ve (serum + CSF) | 1 -ve, but +ve by RT-PCR and NS1 | - | 4 +ve (1 serum, 3 CSF, all IgM negative) | 6/45 confirmed by NS1 detection | - |
| CHIKV | 45 | 45 +ve | - | 1- ne for IgM, but +ve by RT-PCR | - | - | - | - |
| WNV | 10 | 8 +ve s | 3 +ve in CSF | - | - | - | - | 4 confirmed by PRNT |
| JEV | 70 | 61 +ve (serum + CSF) | 5 +ve (serum only), 4 +ve (CSF only) | 47 tested, all but one +ve (both serum and CSF) | - | - | - | - |

**Table S8.** Quality Assessment of Included Studies Using the JBI Critical Appraisal Checklist for Prevalence Studies

| Study (Author, Year) | JBI Score (Yes/9) | JBI Score (%) | Risk of Bias |
| --- | --- | --- | --- |
| Economopoulou (2009) | 7/9 | 77.8 | Moderate |
| Lemant (2008) | 8/9 | 88.9 | Low |
| Bonifay (2018) | 8/9 | 88.9 | Low |
| Thiery (2015) | 6/9 | 66.7 | Moderate |
| Oehler (2015) | 8/9 | 88.9 | Low |
| Tun (2020) | 7/9 | 77.8 | Moderate |
| Sejvar (2005) | 8/9 | 89 | Low |
| Wang (2020) | 8/9 | 89 | low |
| Sebastián (2017) | 8/9 | 89 | Moderate |
| Zambrano (2019) | 6/9 | 66.7 | Moderate |
| Baskar (2018) | 8/9 | 89 | low |
| Del Carpio-Orantes (2020) | 9/9 | 100 | low |
| Parra (2016) | 9/9 | 100 | Low |
| Rozé (2017) | 8/9 | 88.9 | Low |
| Dirlikov (2016) | 7/9 | 77.8 | Moderate |
| Balavoine (2017) | 8/9 | 89 | low |
| Matos (2020) | 6/9 | 66.7 | Moderate |
| Ravi (1994) | 7/9 | 77.8 | Moderate |
| Leonhard (2021) | 8/9 | 88.9 | Low |
| Simon (2018) | 9/9 | 100 | Low |
| Grijalva (2020) | 8/9 | 88.9 | Low |
| Dirlikov (2017) | 8/9 | 88.9 | Low |
| GeurtsvanKessel (2018) | 7/9 | 77.8 | Moderate |
| Cao-Lormeau (2016) | 9/9 | 100 | Low |
| Stegmann-Planchard (2020) | 7/9 | 77.8 | Moderate |
| Dutta (2021) | 7/9 | 77.8 | Moderate |
