# Supplementary material for: Arbovirus‐Associated Guillain–Barré Syndrome: A Systematic Review and Meta‐Analysis of Clinical Characteristics, Subtypes, and Vaccine Associations
Source: Immun Inflamm Dis. 2026 Jul 6;14(7):e70483. doi: 10.1002/iid3.70483 (PMC13338631; doi:10.1002/iid3.70483)
Supplement: Supplementary file 5 — Table S9: PRISMA 2020 checklist for systematic reviews and meta‐analyses. Table S10: Keywords and search strategy used for literature identification. [file IID3-14-e70483-s005.docx]

# Table S10. PRISMA 2020 Checklist – Arbovirus-associated Guillain-Barré Syndrome Meta-Analysis

| **Section and Topic** | **Item #** | **Checklist item** | **Reported in manuscript / ✓** |
| --- | --- | --- | --- |
| TITLE | 1 | Identify the report as a systematic review and/or meta-analysis. | ✓ Title: “A Systematic Review and Meta-Analysis” |
| ABSTRACT | 2 | See PRISMA 2020 for Abstracts checklist. | ✓ Abstract section (Page 1–2) |
| INTRODUCTION | 3 | Describe the rationale for the review in the context of what is already known. | ✓ Introduction (Pages 2–5) |
|  | 4 | Provide an explicit statement of the objectives or questions the review addresses. | ✓ Last paragraph of Introduction (Page 5) |
| METHODS | 5 | Specify inclusion and exclusion criteria and how studies were grouped for syntheses. | ✓ Study Design, Search Strategy and Inclusion Criteria (Pages 5–6) |
|  | 6 | Specify all databases, registers, websites, and other sources searched, including last search date. | ✓ Study Design and Search Strategy (Page 5): PubMed, Scopus, Web of Science, Google Scholar, up to early 2025 |
|  | 7 | Describe the full search strategy for at least one database. | ✓ Supplementary Appendix 1 (Search Strategy) |
|  | 8 | Specify screening methods, number of reviewers, independence, and any automation tools used. | ✓ Data Extraction (Page 6): Two reviewers, consensus or third reviewer |
|  | 9 | Specify methods to collect data, reviewers involved, and processes for confirmation. | ✓ Data Extraction (Page 6) |
|  | 10a | List and define all outcomes sought. | ✓ Data Extraction and Meta-Analysis (Pages 6–7) |
|  | 10b | List and define other variables sought (study characteristics, funding, etc.). | ✓ Data Extraction (Page 6) |
|  | 11 | Specify methods used to assess risk of bias and how used in synthesis. | ✓ Risk of Bias and Quality Assessment (Page 7): JBI Checklists |
|  | 12 | Specify effect measures used (e.g., OR, mean difference). | ✓ Statistical Analysis (Page 7) |
|  | 13a | Describe how studies were selected for each synthesis. | ✓ Study Design, Search Strategy and Inclusion Criteria (Pages 5–6) |
|  | 13b | Describe data preparation methods (handling of missing data). | ✓ Statistical Analysis (Page 7) |
|  | 13c | Describe methods used to tabulate or visualize results. | ✓ Figures 1–9, Tables 1–5, Supplementary Tables S1–S7 |
|  | 13d | Describe synthesis methods and rationale. | ✓ Meta-Analysis (Pages 6–7): Random-effects model using R |
|  | 13e | Describe methods to explore heterogeneity. | ✓ Statistical Analysis (Page 7): I² and subgroup analysis |
|  | 13f | Describe sensitivity analyses conducted. | ✗ Not performed |
|  | 14 | Describe methods to assess publication bias. | ✓ Statistical Analysis (Page 7): Funnel plot asymmetry and regression tests |
|  | 15 | Describe methods to assess certainty in evidence. | ✗ Not assessed |
| RESULTS | 16a | Report search and selection process, ideally with a flow diagram. | ✓ Study Selection (Page 8) and Figure 1 |
|  | 16b | Cite excluded studies and reasons. | ✓ Exclusion criteria in Study Design (Page 6) |
|  | 17 | Cite included studies and describe characteristics. | ✓ Study characteristics (Pages 8–9); Tables 1–3 |
|  | 18 | Present risk of bias for each included study. | ✓ Risk of Bias and Quality Assessment (Page 7) |
|  | 19 | Present results of individual studies for all outcomes. | ✓ Meta-analysis Results (Pages 9–12) |
|  | 20a | Present results of all statistical syntheses. | ✓ Meta-analysis sections (Pages 9–13) |
|  | 20b | Report direction, magnitude, and precision of effects. | ✓ Figures 2–9 |
|  | 20c | Report results of heterogeneity analyses. | ✓ Subgroup analyses (Pages 9–11) |
|  | 20d | Report results of sensitivity analyses. | ✗ Not performed |
|  | 21 | Present assessment of risk of bias due to missing results. | ✓ Statistical Analysis (Page 7): Funnel plots |
|  | 22 | Present assessments of certainty. | ✗ Not assessed |
| DISCUSSION | 23a | Interpret results in context of other evidence. | ✓ Discussion (anticipated) |
|  | 23b | Discuss limitations of included evidence. | ✓ Discussion (anticipated) |
|  | 23c | Discuss limitations of review processes. | ✓ Discussion (anticipated) |
|  | 23d | Discuss implications for practice and research. | ✓ Discussion and Conclusion (anticipated) |
| OTHER INFORMATION | 24a | Provide registration information. | ✗ Not registered |
|  | 24b | Indicate where protocol can be accessed. | ✗ Not applicable |
|  | 24c | Describe amendments to protocol or registration. | ✗ Not applicable |
|  | 25 | Describe funding and support sources. | ✓ Acknowledgments or Funding (anticipated) |
|  | 26 | Describe competing interests. | ✓ Conflict of Interest statement (anticipated) |
|  | 27 | Report availability of data, code, and materials. | ✓ Statistical Analysis (Page 7): R meta/metafor; Data availability not specified |

**Table S10:** Keywords and search strategy used for literature identification.

| **Database** | **Search String** |  |  |
| --- | --- | --- | --- |
| PubMed | ("Guillain-Barré Syndrome" OR "GBS") AND ("arbovirus" OR "arboviral infection" OR "vector-borne virus" OR "mosquito-borne virus") AND ("Zika virus" OR "ZIKV" OR "Dengue virus" OR "DENV" OR "Chikungunya virus" OR "CHIKV" OR "West Nile virus" OR "WNV" OR "Japanese encephalitis virus" OR "JEV" OR "Tick-borne encephalitis virus" OR "TBEV" OR "Toscana virus" OR "Murray Valley encephalitis" OR "Usutu virus") |  |  |
| Scopus | TITLE-ABS-KEY("Guillain-Barré Syndrome" OR "GBS") AND TITLE-ABS-KEY("arbovirus" OR "arboviral infection" OR "vector-borne virus" OR "mosquito-borne virus") AND TITLE-ABS-KEY("Zika virus" OR "Dengue virus" OR "Chikungunya virus" OR "West Nile virus" OR "Japanese encephalitis virus" OR "Tick-borne encephalitis virus" OR "Toscana virus" OR "Murray Valley encephalitis" OR "Usutu virus") |  |  |
| Web of Science | TS=("Guillain-Barré Syndrome" OR "GBS") AND TS=("arbovirus" OR "arboviral infection" OR "vector-borne virus" OR "mosquito-borne virus") AND TS=("Zika virus" OR "Dengue virus" OR "Chikungunya virus" OR "West Nile virus" OR "Japanese encephalitis virus" OR "Tick-borne encephalitis virus" OR "Toscana virus" OR "Murray Valley encephalitis" OR "Usutu virus") |  |  |
| Google Scholar | "Guillain-Barré Syndrome" AND ("arbovirus" OR "arboviral infection" OR "vector-borne virus" OR "mosquito-borne virus") AND ("Zika virus" OR "Dengue virus" OR "Chikungunya virus" OR "West Nile virus" OR "Japanese encephalitis virus" OR "Tick-borne encephalitis virus" OR "Toscana virus" OR "Murray Valley encephalitis" OR "Usutu virus") |  |  |
| Embase | ('Guillain-Barre Syndrome'/exp OR 'GBS') AND ('arbovirus'/exp OR 'arboviral infection' OR 'vector-borne virus' OR 'mosquito-borne virus') AND ('Zika virus'/exp OR 'Dengue virus'/exp OR 'Chikungunya virus'/exp OR 'West Nile virus'/exp OR 'Japanese encephalitis virus'/exp OR 'Tick-borne encephalitis virus'/exp OR 'Toscana virus'/exp OR 'Murray Valley encephalitis'/exp OR 'Usutu virus'/exp) |  |  |
| Cochrane Library | ("Guillain-Barré Syndrome" OR "GBS") AND ("arbovirus" OR "arboviral infection" OR "vector-borne virus" OR "mosquito-borne virus") AND ("Zika virus" OR "Dengue virus" OR "Chikungunya virus" OR "West Nile virus" OR "Japanese encephalitis virus" OR "Tick-borne encephalitis virus" OR "Toscana virus" OR "Murray Valley encephalitis" OR "Usutu virus") |  |  |
